# Anæsthesia

**Published:** 1868-04

**Authors:** A. T. Hudson

**Affiliations:** Lyons, Iowa


### ANÆSTHESIA.

Reported to the Iowa State Medical Society, at its session in Davenport, May, 1867.

BY A. T. HUDSON, M. D., OF LYONS, IOWA.

In the collection of facts from which to draw a few practical observations on the subject of anæsthesia I expected to have received some assistance from the two gentlemen who were appointed on the committee with me to make this report; but hearing nothing from them, I have waited longer for their aid than I ought to have done to leave time for a full or valuable one.

During the twenty years since the discovery of this great boon to suffering humanity several agents have been added to swell the list of anæsthetics. With the advent of each new one, certain qualities of superiority were promised over others, that appeared fair in theory, but often proving worthless in practice.

Of all the articles thus employed none have risen to an equality with chloroform and ether.

In the selection of the best article for anæsthesia, the first thing required is one that affords the most complete immunity from pain during surgical operations, and one that is the most safe and practical in its applications. All agree that chloroform fulfills the first requirement with wonderful certainty. It is easily administered, and rarely, if ever, fails to give the desired relief. The next attribute—that of safety—is yet a mooted question. The opinions of professional men are divided between chloroform and ether. Those who believe ether to be the safest prefer to use it, notwithstanding its frequent slowness and uncertainty; for it will sometimes fail, unless pushed with some degree of severity. Some of its most ardent advocates admit that the only way to get its full effects speedily is by using a saturated sponge within a paper funnel, thereby excluding the air; and when once applied, hold the funnel *remorselessly to the face*, regardless of the patient's struggles, until he is oblivious. If any intermission is allowed for breathing, all advantage is lost. With some patients the laryngeal irritaton is so great that it becomes a fearful ordeal to go through with, scarcely more endurable than the pain of an operation. For these, or other reasons, we see that the use of ether is diminishing, while that of chloroform is extending.

The number of deaths that are now recorded as occurring under chloroform are two hundred, and thirty-six from ether. This is a dark record against chloroform; but when the number of administrations are computed above those of ether, the percentage is about equal. This number of fatal cases, however, has been greatly reduced by careful examination, and the exclusion of all such cases as the following:

"M. Cazenave, of Bordeaux, was called to amputate the leg of a gentleman, aged forty, whose horse had fallen upon the limb. Several hours had elapsed between the accident and the intended operation. The patient was greatly depressed, and made frequent lamentations at the idea of losing his leg. M. Cazenave exhorted him to take courage, and at the patient's request it was agreed that chloform should be used; but as he was so despondent, and the powers of life so low, Cazenave resolved to withhold the chloroform, asking the doctor assisting to feign its administration. The hankerchief was therefore held at a great distance from the face; but the patient had hardly made four hurried inspirations before respiration ceased, and the action of the heart stopped. All efforts at restoration were vain."

From a vague supposition that chloroform increased the mental depression, the anæsthesia is charged as one factor in causing death. Death in this case was undoubtedly from mental shock. When all such are excluded by careful analysis, which has been done by M. Perrin, the actual number of deaths under chloroform is reduced to 109—almost one-half.

Great difference of opinion exists as to the mode of death under chloroform. As long as post mortem reveals nothing to solve the mystery of these forms of death, such differences must ever exist.

It is said by Sansom that death takes place by three modes: by syncope, by asphyxia, and by necremia. Death from syncope is so closely allied to shock that but few, I apprehend, can make a practical distinction. Like shock, it follows mental emotion, nervous irritation, and physical injury.

Asphyxia occurs in consequence of imperfect interchange of oxygen and carbonic acid gas, thereby causing congestion from impure blood. Hence death would rarely occur, in this way, only from the prolonged duration of the anæsthetic. In this form of death the countenance is generally pale; the blood is not black, the veins not distended; showing a pathological state opposite to that of true asphyxia. It is therefore a spurious kind of asphyxia, and should be so denominated.

Death by necremea he says "is due to a faulty condition of the circulating blood, which enfeebles the heart, impairs the action of the nervous system, and prevents the due performance of vital changes in the systemic capiliaries." What else is asphyxia but a faulty condition of the blood, working the same changes?

Necremea means impoverished blood, or death of blood; hence a pathological condition of such slow growth that it is difficult to see how it could be induced in the short time that a patient would be under chloroform. This last explanation of the mode of death is not well made out, and therefore is unsatisfactory.

It has been my fortune to witness two deaths under chloroform. The first was a man aged 65. He was to undergo excision of a large tumor of the thigh. He came under the influence very fairly, and continued for fifteen or twenty minutes, when he began to rouse from the stupor and exhibit evidence of pain and consciousness, when more chloroform was directed to complete the operation. At this juncture the physician giving the chloroform was called to assist in the ligation of an artery, and the napkin was entrusted to a non-medical assistant. Immediately after the application of the last chloroform his respiration became difficult and stertorous, and in a few moments ceased altogether. No post mortem was had. The sudden and incautious application of a large volume of vapor was presumed to have been the cause of death.

The second was a soldier wounded in the hand. He was brought to the field hospital during the siege of Atlanta. He was a strong, plethoric man, about 30 years old A chloroform napkin was put to his face in the usual way, by an assistant, when not more than a half dozen inspirations were made before spasmodic, stertorous breathing announced the fatal shock. The surgeon, who was about to operate, sprung to his relief, dashed water in his face, and tried artificial respiration, but he died in a few moments. A careful post mortem revealed nothing to solve the hidden cause of death, unless we should add a diathesis strongly predisposed to apoplexy. Contrary to our usual custom, stimulants were not given prior to the chloroform.

According to an able review of different writers upon this subject in the *American Journal of Medical Sciences*, to which I am indebted for many valuable facts, the accepted doctrine now is, that death takes place by syncope, or paralysis of the heart, and asphyxia. Under the head of syncope are included nearly all sudden deaths—those from emotional causes and from organic origin. Idiosyncracy, which was at one time included, is now abandoned.

Sederation is a term applied by the French to this form of death. The definition given of this is, "sudden death, instantaneous, without material cause, the result of some dynamic perturbation of the entire nervous system, as well as the nerves of the periphery, as their central point of emergence." Such new terms, with their profoundly scientific explanation, serve to confound rather than enlighten. Everything is expressed in the first line, "sudden death, without material cause." Beyond this we are in the dark.

As these different terms, sederation, cardiac paralysis, syncope, and shock, are all used synonymously to express the same form of death, much would be gained in adopting the plainest, simplest, and the most significant one. The word shock is plain, comprehensive, and appropriate.

Under this head, with a single subdivision, may be classed all the deaths that follow when little or no chloroform has been used, leaving but few to be accounted for under the head of spurious asphyxia.

The first subdivision would embrace all deaths after chloroform, combined with emotional causes, prior to operations. Second, those that follow sensory influences during and after operations.

The soldier who died in the field hospital, before mentioned, illustrates the first class. By comparing it with the following cases of undoubted shock, where chloroform was not used, the analogy will be observed.

"Deasault was about to operate upon a patient bound up for lithotomy. He traced with his finger nail the line of incision in the perineum. The man shrieked and died."

M. Perrin says: "A soldier, aged 25, affected with phymosis, with concealed fungous growth, submitted very reluctantly to an operation. No sooner did he see the surgeon's hand, armed with a bistory, than he fell into a syncope, and died instantly."

"An old man, who was suspected of having stone in the bladder, was to be sounded. He had an unconquerable fear of this operation. Scarcely had the surgeon approached the meatus with the sound than, seized with terror, he died in the presence and in spite of the earnest care of such celebrated practitioners as Civiale and Honore." The emotion of fright and apprehension undoubtedly caused the shock that produced death. These cases represent so nearly the type of those that occur after a few breaths of chloroform, that it is folly to class them differently.

The following cases bear the same typical analogy to the second division, or those that take place during and after operations where the sensory system, especially the cutaneous surface, is involved.

"A woman 45 years old, of bilious temperament, and very irritable, had a cancer of the breast. She reluctantly consented to an operation. Just as the surgeon had made two incisions, and before the tumor could be dissected out, she died upon his hands." Two such accidents happened under the hand of Reaux.

"Listor lost a patient, who started and died as a single sweep of the knife amputated the penis."

"Dr. Mackenzie being called to see a man who had fractured his radius, had some thought of employing chloroform in making the examination, but changed his mind, and made the necessary manipulations without it. He proceeded to leave the house, but had scarcely got down the steps when he was called back with the announcement that his patient had suddenly died."

Many other cases could be cited, but these are sufficient to show some of the forms of death by the shock upon nervous centres and the line of parallel they bare to those under chloroform.

Several deaths have followed the very limited use thus far made of nitrous oxyde gas and amelin. And such accidents may be expected to occur with any and all anæsthetics which may be extensively used, so long as their danger lies more in the manner of administration than in any supposed noxious atributes of the agents. When we reflect how frequently such sudden deaths have occurred in ordinary practice, need we be surprised that they should continue to follow in about the same ratio as before the era of anæsthesia? I think not, and so, likewise, do eminent men think, who have examined this subject attentively.

The power of chloroform to hasten reaction, when the heart and nerve forces are nearly overpowered by the shock of physical injury, is now generally acknowledged. Owing to this valuable attribute many lives have been saved that would otherwise have been lost by delay. A case demonstrating well this fact occurred after a battle near Macon, Georgia. A soldier having his knee badly torn in pieces by a shell, was placed upon one of the tables for amputation. His feeble pulse, pallor, coldness, and apparent exhaustion, indicated speedy dissolution. The two operating surgeons in consultation with the surgeon in chief decided that he would die soon, and very likely during the amputation, if one was attempted. He was therefore laid back for reaction, or to die. A few minutes after he was put upon another table by two surgeons, who were not aware that any examination or decision had been made upon him. They recognized the desperate nature of the case, and hesitated about proceeding; but believing that the only hope lay in speedy amputation, they gave a gill of whisky, followed soon after by chloroform, and the operation was done. When it was over his pulse indicated an effort at reaction. He was put into an ambulance the next morning, and afterwards carried two hundred miles, and he made a good recovery.

It is a noteworthy fact that chloroform rarely, if ever, produces death by a poisonous, or what might be called an over dose. If it was uniformly so, we should have a class of characteristic symptoms as clearly marked as the spasm from strychnia, or the coma from opium; but such is not the case, and the idea is refuted by the singular fact that far the greater number of deaths have followed when the smallest amount of the article has been given, and then upon the most robust and healthy patients, and when least to be anticipated.

The rate of mortality of capital operations compared with minor ones, or such as belong to dental practice are 10 to 100.

A writer upon this subject assigns a reason for this great difference, which is, that in trivial cases complete anæsthesia is not often reached, leaving the mind in a semi-unconscious state, particularly dangerous when the shock of pain is added to an over strained nervous system. Total anæsthesia carries the system beyond this state, and out of danger, "hence the conclusion that total anæsthesia is better than partial."

Now, what shall be said of the treatment of that state of the system which so quickly ends in death under chloroform?

If there are any prophylactic or therapeutic means known which are serviceable in the ordinary forms of shock, syncope, and asphyxia, then by the proper application in parallel cases under anæsthesia many lives may be saved.

The means hitherto employed, when a patient sinks with alarming symptoms, are cold water dashed in the face, whipping or slapping the surface and extremities, artificial respiration and galvanism—all energetic stimulants—and in such a crisis we would gladly add brandy; but unfortunately it cannot be swallowed, and, powerless and sad, we stand by and see life extinguished for want of it.

A surgeon who has ever witnessed such a death would never forget how ardently he wished and prayed for power to introduce an ounce of brandy the instant he saw the countenance blanch and the breathing and pulse begin to fail.

Was there ever an instance known of a person being seized with a faint, or nervous shock of any kind, when under high exhileration of stimulants? On the contrary they have been universally used to obtund the sensibilities and calm apprehension.

The prophylactic value of the pro-exhibition of stimulants in such cases is in accordance with sound principles and good practice.

A measure to anticipate or provide against the dangers of anæsthesia has recently been recommended by a chloroform committee of London. It is to mix alcohol and ether with chloroform. So far as this has been tried it meets with considerable favor; yet one case of death is reported, occurring after the administration of the mixture. This need not surprise us. The reason appears obvious. If stimulants are valuable, they must be given in sufficient quantities to answer the end desired. In the mixture the amount is too small. If this is a step in the right direction, why not advance boldly to the goal of safety, and give the requisite amount required to keep the heart and nervous centres steady. Alcohol being a supporter of respiration, will relieve, or give protection from the other sources of danger which follow deficient action, and ends in asphyxia.

For twelve years it has been my custom to give stimulants prior to chloroform. Since adopting this plan I have never witnessed an alarming symptom. This I am aware is negative testimony, of not much value; but I have searched the records of fatal cases for a single one where alcohol had preceded the anæsthesia, but without success.

In our division of the army chloroform was almost exclusively used, and as a rule was always preceded with whisky, although it was occasionally omitted, as was the case of the soldier already noticed.

Aside from its prophylactic value, there is a great economy of time and of material, and never failure in the desired result.

Much has been said and anticipated from Dr. Richardson's plan of local anæsthesia. It offers one positive advantage over all inhalations, which is that it is perfectly safe. It is a very handsome thing in theory, but does not fulfill all expectations in practice. Others may be more fortunate in its use, and think well of it; but from my own limited experience, it does not give entire protection from pain during operations. It seems to be quite inadequate to afford insensibility to the deeper structure, therefore the throbbing pain of chill is added to that of the knife.

Patients say that the pain of freezing is equally distressing as that of cutting.

If it fails in the most essential particular—complete immunity from pain—then the crowning glory of anæsthesia is gone.

The following practical conclusions make up a summary of this subject:

Of the different anæsthetic agents now in use chloroform is generally employed, and is the best. A napkin is better than any instrument yet invented for inhalation. Its chief danger lies in its too rapid and incautious administration..

No morbid state of the system, as diseased heart, lungs, liver, or blood, forbid its use, unless delirium tremens is excepted.

That all who are able to undergo an operation are proper subjects.

There are no indications to foreshadow disaster and death.

That the greatest rate of mortality is with the strong and robust, and females less liable to danger than males; and children rarely, if ever, die under the effects of chloroform.

That full anæsthesia is better and safer than partial.

The fact of having taken it before is no evidence of security thereafter.

It will diminish the shock of injury, and may be given without regard to the time of reaction.

And that stimulants should always be given prior to inhalation.

---

It has been my privilege to administer anæsthetics for twenty years; for fifteen years have occupied the chair of surgery in the Medical Department of the Iowa University. My preference has been for chloroform unadulterated; have but in a single instance witnessed its injurious effects, and have rarely preceded its use by stimulants. As a local anæsthetic the rigoline promises advantages over that of ether, being more rapid in its effects, and consequently less suffering in its application. Ed.

